# Supplementary material for: Calcium handling precedes cardiac differentiation to initiate the first heartbeat
Source: eLife. 2016 Oct 11;5:e17113. doi: 10.7554/eLife.17113 (PMC5059139; doi:10.7554/eLife.17113)
Supplement: Supplementary file 1. — (a) Morphological criteria of stages of cardiac crescent development. Different stages of cardiac crescent development (stage 0 to stage 3) were defined based on the ratio between the weight and maximum width of the cardiac crescent. As development progresses the width decreases and the maximum height increases. These stages are a more accurate representation of developmental stage than more widely used staging criteria such as somite number and embryonic day. (b) Statistical comparison of qRT-PCR results of isolated cardiac crescents. Table of p-values obtained from ANOVA and a post-hoc Tukey test on the qRT-PCR results obtained from isolated cardiac crescents, comparing whole embryos pre-cardiac crescent formation (E7.75), at all stages of cardiac crescent development (stage 0 to stage 3) and linear heart tube (LHT) stage. (c) Statistical comparison of qRT-PCR results of isolated head folds. Table of p-values obtained from ANOVA and a post-hoc Tukey test on the qRT-PCR results obtained from isolated head folds, comparing whole embryos pre-cardiac crescent formation (E7.75), at all stages of cardiac crescent development (stage 0 to stage 3) and linear heart tube (LHT) stage. (d) Analysis from SACO inhibition experiments at Stage 0. Table of results detailing individual inhibitor experiments carried out on SACOs at stage 0. Information includes embryo ID, inhibitor used, absolute number of SACOs observed, before and after treatment, ratio of SACOs maintained after treatment, percentage inhibition, area containing SACOs and length of imaging. (e) List of antibodies. List of primary antibodies used for immunostaining and western blot analyses and secondary antibodies used for all the experiments with source and dilution used. (f) List of primers for qRT-PCR. List of primer pairs used for qRT-PCR analysis and respective amplicon size. DOI: http://dx.doi.org/10.7554/eLife.17113.024 [file elife-17113-supp1.docx]

**Supplementary file 1a.** Embryo staging specific to the early developing heart from cardiac crescent to linear heart tube.

| **Stage** | **Width** | **Height** | **Width/Height Ratio** | **Somites** | **Embryonic Day** |
| --- | --- | --- | --- | --- | --- |
| 0 | 360-390 | 70-80 | 4.5-5.5 | 0-2 | E7.75-E8.0 |
| 1 | 300-370 | 70-95 | 3.0-4.2 | 1-3 | E8.0-E8.25 |
| 2 | 230-280 | 95-120 | 2.0-2.8 | 2-4 | E8.0-E8.5 |
| 3 | 200-230 | 120-140 | 1.5-1.8 | 3-5 | E8.25-E8.5 |

**Supplementary file 1b.** ECC component gene expression assessed by qRT-PCR in embryonic hearts from stage 0-LHT stages: relative p-values shown arising from one-way ANOVA and Tukey test for multiple comparisons.

| **Comparison** | ***Slc8a1*** | ***Cacna1c*** | ***Cacna1d*** | ***Cacna1g*** | ***Cacna1h*** | ***Ryr2*** | ***Atp2a2*** | ***Itpr2*** | ***Nkx2.5*** | ***Tnnt2*** | ***Actn2*** | ***Myom1*** | ***Hcn4*** |
| --- | --- | --- | --- | --- | --- | --- | --- | --- | --- | --- | --- | --- | --- |
| **E7.75 - Stage 0** | 0.0002 | 0.2635 | 0.0078 | 0.0583 | 0.9989 | 0.0006 | 0.0241 | 0.3823 | 0.0000 | 0.0958 | 0.5653 | 0.3338 | 0.0000 |
| **Stage 0 - Stage 1** | 0.0000 | 0.0004 | 0.0000 | 0.7020 | 0.1108 | 0.1056 | 0.0431 | 0.1723 | 0.1275 | 0.0248 | 0.0128 | 0.0216 | 0.0002 |
| **Stage 1 - Stage 2** | 0.0000 | 0.0007 | 0.0009 | 0.8632 | 0.0097 | 0.1516 | 0.2717 | 0.9019 | 0.6776 | 0.0909 | 0.0253 | 0.0240 | 0.2231 |
| **Stage 2 - Stage 3** | 0.0088 | 0.0001 | 0.0119 | 0.4843 | 0.0001 | 0.4498 | 0.9133 | 0.3918 | 0.8682 | 0.0430 | 0.0349 | 0.0088 | 0.3163 |
| **Stage 3 - LHT** | 0.9170 | 0.9526 | 0.0267 | 0.9386 | 0.0229 | 0.9933 | 0.8850 | 0.9998 | 0.4589 | 0.6662 | 0.9320 | 0.9997 | 0.4548 |
|  |  |  |  |  |  |  |  |  |  |  |  |  |  |
| **E7.75 - Stage 1** | 0.0000 | 0.0000 | 0.0000 | 0.0020 | 0.2198 | 0.0000 | 0.0000 | 0.9960 | 0.0000 | 0.0000 | 0.0002 | 0.0001 | 0.0000 |
| **Stage 0 - Stage 2** | 0.0000 | 0.0000 | 0.0000 | 0.9996 | 0.0000 | 0.0003 | 0.0002 | 0.0199 | 0.0047 | 0.0000 | 0.0000 | 0.0000 | 0.0000 |
| **Stage 1 - Stage 3** | 0.0000 | 0.0000 | 0.0000 | 0.0701 | 0.0000 | 0.0000 | 0.0390 | 0.0614 | 0.1350 | 0.0000 | 0.0000 | 0.0000 | 0.0022 |
| **Stage 2 - LHT** | 0.0008 | 0.0009 | 0.9992 | 0.1087 | 0.0000 | 0.7776 | 1.0000 | 0.2623 | 0.9772 | 0.5698 | 0.0039 | 0.0044 | 0.9998 |
|  |  |  |  |  |  |  |  |  |  |  |  |  |  |
| **E7.75 - Stage 2** | 0.0000 | 0.0000 | 0.0000 | 0.0296 | 0.0000 | 0.0000 | 0.0000 | 0.6518 | 0.0000 | 0.0000 | 0.0000 | 0.0000 | 0.0000 |
| **Stage 0 - Stage 3** | 0.0000 | 0.0000 | 0.0000 | 0.6736 | 0.0000 | 0.0000 | 0.0000 | 0.0002 | 0.0003 | 0.0000 | 0.0000 | 0.0000 | 0.0000 |
| **Stage 1 - LHT** | 0.0000 | 0.0000 | 0.0004 | 0.0089 | 0.0000 | 0.0089 | 0.3074 | 0.0345 | 0.9716 | 0.0020 | 0.0000 | 0.0000 | 0.1402 |
|  |  |  |  |  |  |  |  |  |  |  |  |  |  |
| **E7.75 - Stage 3** | 0.0000 | 0.0000 | 0.0000 | 0.6492 | 0.0000 | 0.0000 | 0.0000 | 0.0207 | 0.0000 | 0.0000 | 0.0000 | 0.0000 | 0.0000 |
| **Stage 0 - LHT** | 0.0000 | 0.0000 | 0.0000 | 0.1939 | 0.0000 | 0.0000 | 0.0003 | 0.0001 | 0.0256 | 0.0000 | 0.0000 | 0.0000 | 0.0000 |
|  |  |  |  |  |  |  |  |  |  |  |  |  |  |
| **E7.75 - LHT** | 0.0000 | 0.0000 | 0.0000 | 0.9889 | 0.0000 | 0.0000 | 0.0000 | 0.0112 | 0.0000 | 0.0000 | 0.0000 | 0.0000 | 0.0000 |
|  |  |  |  |  |  |  |  |  |  |  |  |  |  |
| **Significance** | ******* | ****** | ***** | **NS** |  |  |  |  |  |  |  |  |  |

**Supplementary file 1c.** Gene expression analysed by qRT-PCR in isolated head folds from embryos at E7.75 to LHT stages: relative p-values shown arising from one-way ANOVA and Tukey test for multiple comparisons.

| **Comparison** | ***Sox1*** | ***Cacna1c*** | ***Slc8a1*** |  |
| --- | --- | --- | --- | --- |
| **E7.75 - Stage 0** | 0.0003 | 0.1077 | 1.0000 |  |
| **Stage 0 - Stage 1** | 0.0177 | 1.0000 | 0.9422 |  |
| **Stage 1 - Stage 2** | 0.8794 | 0.9917 | 0.9691 |  |
| **Stage 2 - Stage 3** | 0.9213 | 0.9909 | 1.0000 |  |
| **Stage 3 - LHT** | 0.9993 | 1.0000 | 0.9453 |  |
|  |  |  |  |  |
| **E7.75 - Stage 1** | 0.0000 | 0.1537 | 0.9466 |  |
| **Stage 0 - Stage 2** | 0.0013 | 0.9712 | 1.0000 |  |
| **Stage 1 - Stage 3** | 1.0000 | 0.8491 | 0.9846 |  |
| **Stage 2 - LHT** | 0.7710 | 0.9864 | 0.9692 |  |
|  |  |  |  |  |
| **E7.75 - Stage 2** | 0.0000 | 0.3939 | 1.0000 |  |
| **Stage 0 - Stage 3** | 0.0134 | 0.7548 | 0.9999 |  |
| **Stage 1 - LHT** | 0.9999 | 0.8235 | 0.6369 |  |
|  |  |  |  |  |
| **E7.75 - Stage 3** | 0.0000 | 0.7437 | 0.9999 |  |
| **Stage 0 - LHT** | 0.0297 | 0.7237 | 0.9859 |  |
|  |  |  |  |  |
| **E7.75 - LHT** | 0.0000 | 0.7739 | 0.9848 |  |
|  |  |  |  |  |
| **Significance** | ******* | ****** | ***** | **NS** |

**Supplementary file 1d.** Stage 0 embryo drug treatments; documenting degree of inhibition of SACOs with area and duration of image capture in the early cardiac crescent.

| **Embryo** | **Drug** | **SACO number** | | **Ratio** | **% Inhibited** | **Area Imaged (µm^2^)** | **Duration (sec)** |
| --- | --- | --- | --- | --- | --- | --- | --- |
|  |  | **Baseline** | **15 Min Drug** |  |  |  |  |
| Exp7E2 | DMSO | 14 | 12 | 0.8571 | 14.3% | 16257.43 | 19.73 |
| Exp7E3 | DMSO | 11 | 9 | 0.8182 | 18.2% | 10031.30 | 19.73 |
| Exp7E6 | DMSO | 6 | 5 | 0.8333 | 16.7% | 8112.17 | 19.73 |
| Exp7E7 | DMSO | 7 | 6 | 0.8571 | 14.3% | 5564.36 | 19.73 |
| Exp7E8 | DMSO | 14 | 9 | 0.6429 | 35.7% | 11239.02 | 19.73 |
| Exp8E1 | DMSO | 7 | 5 | 0.7143 | 28.6% | 3912.70 | 19.73 |
| Exp8E3 | DMSO | 6 | 2 | 0.3333 | 66.7% | 5172.57 | 19.73 |
| Exp8E5 | DMSO | 14 | 7 | 0.5000 | 50.0% | 14699.52 | 19.73 |
| Exp13E1 | DMSO | 11 | 11 | 1.0000 | 0.0% | 6256.46 | 16.72 |
| Exp13E5 | DMSO | 7 | 4 | 0.5714 | 42.9% | 3077.22 | 16.72 |
| Exp6E2 | CB-DMB | 14 | 6 | 0.4286 | 57.1% | 14429.45 | 19.73 |
| Exp6E3 | CB-DMB | 6 | 0 | 0.0000 | 100.0% | 6524.25 | 19.73 |
| Exp6E4 | CB-DMB | 12 | 0 | 0.0000 | 100.0% | 9842.20 | 19.73 |
| Exp6E6 | CB-DMB | 5 | 0 | 0.0000 | 100.0% | 3397.71 | 19.73 |
| Exp6E12 | CB-DMB | 6 | 0 | 0.0000 | 100.0% | 3507.10 | 19.73 |
| Exp6E14 | CB-DMB | 8 | 3 | 0.3750 | 62.5% | 3172.11 | 19.73 |
| Exp11E5 | CB-DMB | 9 | 0 | 0.0000 | 100.0% | 5000.00 | 16.72 |
| Exp11E7 | CB-DMB | 6 | 0 | 0.0000 | 100.0% | 2247.25 | 16.72 |
| Exp9E2 | KB-R7943 | 9 | 0 | 0.0000 | 100.0% | 5037.31 | 16.72 |
| Exp9E3 | KB-R7943 | 14 | 0 | 0.0000 | 100.0% | 5920.06 | 16.72 |
| Exp10E1 | KB-R7943 | 12 | 0 | 0.0000 | 100.0% | 3976.12 | 16.72 |
| Exp10E2 | KB-R7943 | 6 | 2 | 0.3333 | 66.7% | 4513.81 | 16.72 |
| Exp12E1 | KB-R7943 | 16 | 0 | 0.0000 | 100.0% | 7320.80 | 16.72 |
| Exp12E2 | KB-R7943 | 5 | 0 | 0.0000 | 100.0% | 1979.79 | 16.72 |
| Exp12E3 | KB-R7943 | 7 | 0 | 0.0000 | 100.0% | 5420.98 | 16.72 |
| Exp12E4 | KB-R7943 | 6 | 0 | 0.0000 | 100.0% | 2823.54 | 16.72 |
| Exp12E6 | KB-R7943 | 4 | 2 | 0.5000 | 50.0% | 2551.49 | 16.72 |
| Exp4E3 | Nifedipine | 7 | 6 | 0.8571 | 14.3% | 7611.24 | 19.73 |
| Exp4E5 | Nifedipine | 24 | 18 | 0.7500 | 25.0% | 17200.49 | 19.73 |
| Exp5E1 | Nifedipine | 14 | 10 | 0.7143 | 28.6% | 9359.09 | 19.73 |
| Exp5E2 | Nifedipine | 8 | 0 | 0.0000 | 100.0% | 4812.86 | 19.73 |
| Exp3E6 | Nifedipine | 9 | 7 | 0.7778 | 22.2% | 6011.51 | 19.73 |
| Exp3E7 | Nifedipine | 13 | 5 | 0.3846 | 61.5% | 4530.28 | 19.73 |
| Exp2E2 | Nifedipine | 14 | 6 | 0.4286 | 57.1% | 6923.74 | 19.73 |
| Exp1E1 | Nifedipine | 11 | 11 | 1.0000 | 0.0% | 10320.82 | 19.73 |

**Supplementary file 1e.** List of primary and secondary antibodies used with working dilutions.

| **Primary Antibody** | **Company** | **Dilution** |
| --- | --- | --- |
| Mouse anti-cTnT | Abcam: ab8295 | 1:100 |
| Rabbit anti-NCX1 | Swant: π11-13 | 1:200 |
| Rabbit anti-Cav1.2 | Santa Cruz: sc-25686 | 1:100 |
| Goat anti-Nkx2.5 | Santa Cruz: sc-8697 | 1:100 |
| Mouse anti-Myom1 | Gift from Dr E. Ehler | 1:10 |
| Rabbit anti-Sarcomeric Alpha Actinin | Abcam: ab68167 | 1:100 |
| Rabbit anti-pCamKII | Abcam | 1:500 |
| Rabbit anti-CamKII | Santa Cruz | 1:500 |
| Mouse anti-GAPDH | Abcam | 1:10000 |
| **Secondary Antibodies** | **Company** | **Dilution** |
| Goat anti-mouse 488 | Life Technologies | 1:200 |
| Goat anti-rabbit 488 | Life Technologies | 1:200 |
| Goat anti-mouse 594 | Life Technologies | 1:200 |
| Goat anti-rabbit 594 | Life Technologies | 1:200 |
| Donkey anti-mouse 555 | Thermo Fisher Scientific: A-31570 | 1:100 |
| Donkey anti-Rabbit 647 | Thermo Fisher Scientific: A-31573 | 1:100 |
| Donkey anti-Goat 488 | Thermo Fisher Scientific: A-11055 | 1:100 |
|  |  |  |

**Supplementary file 1f.** List of primer sets for genes analysed by RT-qPCR.

| **Gene** | **Forward Primer (5’-3’)** | **Reverse Primer (5’-3’)** | **Amplicon (bp)** |
| --- | --- | --- | --- |
| **Gapdh** | AGGTCGGTGTGAACGGATTTG | TGTAGACCATGTAGTTGAGGTCA | 101 |
| **Hprt** | TCAGTCAACGGGGGACATAAA | GGGGCTGTACTGCTTAACCAG | 122 |
| **18s** | GCCGCTAGAGGTGAAATTCTTG | GAAAACATTCTTGGCAAATGCTTT | 71 |
| **Oct-4** | TGGAGGAAGCCGACAACAATGAGA | TGGCGATGTGAGTGATCTGCTGTA | 148 |
| **Brachyury** | CATGCTGCCTGTGAGTCATAA | TGTCTGGGAGCCTGGGGTGAT | 83 |
| **Mesp1** | GTCTGCAGCGGGGTGTCGTG | CGGCGGCGTCCAGGTTTCTA | 189 |
| **Nkx2.5** | CTATGCCCTGTCCCTCAGAT | CTCCCGGTCCTAGTGTGGAA | 139 |
| **Mef2c** | ACTGGGAAACCCCAATCTTC | ATCAGACCGCCTGTGTTACC | 92 |
| **Myh6** | GCTGGCTGGAAAAGAACAAG | TCTTGCCTCCTTTGCCTTTA | 125 |
| **Myh7** | ACTGTCAACACTAAGAGGGTCA | TTGGATGATTTGATCTTCCAGGG | 91 |
| **Tnnt2** | CAGAGGAGGCCAACGTAGAAG | CTCCATCGGGGATCTTGGGT | 119 |
| **Myom1** | GGCCATTCGACTCTTGTTCTC | AATACTCAGCGAAATGTGGGC | 112 |
| **Actn2** | GGGCTATGAGGAGTGGCTATT | AGTCCTTCTGCAGCAAGATCT | 137 |
| **Slc8a1** | GGACCAACAGCTGGAGAGAG | GGCAAACAGAACCTTCCAGA | 152 |
| **Cacna1d** | AGAACACCACGATTGCCCTA | CTTCTGGCTCGTCATCTTGC | 105 |
| **Cacna1c** | GGAGGGCGTGCATAAGCATT | AGGAAGAGATACTCCACTCGTTC | 160 |
| **Cacna1g** | TGTCTCCGCACGGTCTGTAA | AGATACCCAAAGCGACCATCTT | 184 |
| **Cacna1h** | CGTGACACTGGGCATGTTC | CCACCATCTTGATAACCATCTCC | 106 |
| **Ryr2** | ATTATGAAGGTGGTGCCGTATCA | TTCCACTCCACGCGACTCTTA | 88 |
| **Atp2a2** | TGGGCAAAGTGTATCGACAG | CAGCAGGAACTTTGTCACCA | 108 |
| **Sox1** | GGCCGAGTGGAAGGTCATGT | TCCGGGTGTTCCTTCATGTG | 92 |
| **Hcn4** | TCATCTCCTCCATCCCTGTC | CTGGCCAGGTCATAGGTCAT | 202 |
| **Itp3r2** | TCATCATCGCCTTGATTCTG | CAGTTCCCTGGGTCTCATGT | 170 |
| **Camk2d** | CTGGCACACCTGGGTATCTT | ATCCCAGAAGGGTGGGTATC | 125 |
